# Inequalities in Oral Health for Children with Disabilities: A French National Survey in Special Schools

**DOI:** 10.1371/journal.pone.0002564

**Published:** 2008-06-25

**Authors:** Martine Hennequin, Véronique Moysan, Didier Jourdan, Martine Dorin, Emmanuel Nicolas

**Affiliations:** 1 University of Auvergne (Clermont 1) EA 3847, Clermont-Ferrand, France; 2 CHU de Clermont-Ferrand, Clermont-Ferrand, France; 3 Caisse Nationale de l'Assurance Maladie des Travailleurs Salariés (CNAMTS), Paris, France; 4 Institut Universitaire de Formation des Maîtres, JE 2432, Clermont-Ferrand, France; University of Toronto, Canada

## Abstract

**Background:**

Despite wide recognition that children with disability often have poor oral health, few high quality, controlled results are available.

**Method:**

Twenty-four objective and subjective criteria covering feeding, autonomy, access to dental care, oral hygiene, oral disease, general health and behavior were evaluated in a observational cross-sectional study of 2,487 children with disability (DC group), 4,772 adolescents with disability (DA group) and 1,641 children without disability (NDC group). Five algorithms ranked the subjects according to clinical criteria in three original oral health indices: the Clinical Oral Health Index (COHI), indicating the level of oral health problems, the Clinical Oral Care Needs Index (COCNI) giving dental care need levels, and the Clinical Oral Prevention Index (COPI) determining possible needs in terms of dental education initiatives.

**Results:**

DC-group children presented poorer oral health and had greater needs in both treatment and preventive oral health actions than NDC-group children (OR = 3.97, 95% CI = 3.25–4.86 for COHI; OR = 2.01, 95% CI = 1.77–2.28 for COCNI; OR = 5.25, 95% CI = 4.55–6.02 for COPI). These conditions were worse again in the DA group comparing to the DC group (OR = 3.52, 95% CI = 2.7–4.6 for COHI; OR = 1.52, 95% CI = 1.38–1.69 for COCNI; OR = 1.53, 95% CI = 1.39–1.69 for COPI).

**Conclusion:**

Clinical indices generated by algorithmic association of various clinical indicators allow sensitive clinical measurement, and in this study demonstrated inequalities in oral health for children with disabilities schooling in institutions. Questions need now to be addressed as to the measures that could be taken to compensate for this situation.

## Introduction

Oral health is often considered as a probable source of health inequalities in persons with neuromotor and mental deficiencies. The quality of the evidence base supporting this assumption is not high despite numerous studies reporting poor oral health in patient groups with disabilities. Many studies have no control group, use inappropriate indicators or report a partial evaluation of oral health. The 2001 National Survey of Children with Special Health Care Needs conducted in the USA showed that dental care was the most commonly-reported unmet service need [1]. However, none of the clinical data collected through a large sample was able to confirm the parents' declarations. Some studies have aimed to measure only the prevalence of infectious disease, such as carious process or periodontal disease [2]–[4], while others have focused on the occurrence of anatomical deficiencies [5]–[7], traumatic disease [8] or functional incapacity [9]–[12]. Oral health is, however, a far more complex concept that encompasses all aspects of health related to the mouth, the jaw, the teeth, the throat and related tissues [13], [14]. Self-rated questionnaires on oral health-related quality of life aim to offer a global measure of the concept of health but their use is not feasible by the majority of people with intellectual disability [15]–[17]. Proxy questionnaires have been developed for use with persons presenting special medical conditions [18], [19], but they remain too specific to be used universally across all disability groups. The lack of appropriate indicators has consequently made it impossible to compare oral health between groups with and without disabilities. This study was thus designed to provide epidemiological data on oral health gathered using original indicators from a representative sample of children and adolescents with disabilities in France.

## Method

### Database setting

**Figure 1:**
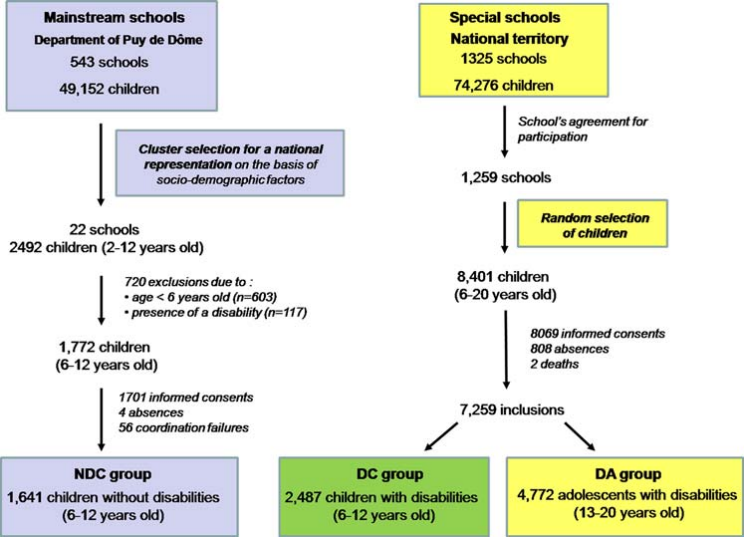
Design for data base setting.

This observational cross-sectional study compared data gathered in the same conditions through two surveys. The design for data base setting is shown in Figure 1. One dataset was taken from a national survey that evaluated oral health in a sample of 6 to 20-year-old children and adolescents in special schooling. The second dataset was taken from a regional survey of a nationally representative sample of 6 to 12-year-old children without disabilities in mainstream schooling. The database thus included three groups differentiated by both age and presence of a disability: one group comprising of 6 to 12-year-old children without disability (NDC group), a second group made up of 6 to 12-year-old children with disability (DC group), and a third group comprising of 13 to 20-year-old adolescents and young adults with disability (DA group). Consent for participation was obtained from the school directors, class teachers, parents, and adult subjects. All legal conditions for epidemiological surveys were respected, and the French national commission governing the application of data privacy laws (the ‘*Commission Nationale Informatique et Libertés*’) issued approval for both projects.

#### Children, adolescents and young adults with disability

The sample populations of children and adolescents with disability were derived from the databases of three national health insurance systems. The population comprised of all 74,276 children, adolescents and young adults born between the 1^st^ October 1984 and the 31^st^ January 1999 attending one of the 1,325 special schools registered in France. The sample size was calculated to determine the results with a 99% Confidence Interval and a precision of ±1.5% around the calculated value. Theoretical national sample size thus reached 7,373 statistical units. Since the risk of non-response was evaluated at between 25% and 35%, the sample size was raised to 10,000 statistical units. For random selection, all children born on a 4^th^, 7^th^, 21^st^ or 27^th^ day of the month were included. The survey was conducted between October 2004 and January 2005.

#### Children without disabilities

The sample of children without disabilities comprised the entire population of 2 to 12-year-old healthy children attending schools situated in the department of Puy de Dôme. This group was devised to be nationally representative, by performing a cluster evaluation design with the school as the intervention unit. The schools were selected on the basis of the following criteria: rural or urban location, socioeconomic status (4 standard groups from underprivileged to highly privileged) and size (more or less than 4 classes) in two school districts (corresponding to a population of 106,088 inhabitants). Twenty two schools were sampled based on these criteria. Comparison between the sample and national data [20] is showed in Table 1. Children under 6 years old and children who received special schooling time for medical reasons were not included in the database. The survey was conducted between January and May 2005.

**Table 1:** Characteristics (expressed as %) of the sample compared to national data [20].

	Location of the schools		Socio-economic status				Number of classes in the school	
	rural	urban	1	2	3	4	<4 classes	>4 classes
**Sample**	54.6%	45.4%	13.0%	17.7%	26.8%	42.2%	40.9%	59.1%
**France (#)**	47.0%	53%	17.9%	14.1%	26.6%	41.4%	41.4%	58.6%

The location is based on the INSEE (National Institute for Statistics and Economic Studies) definition (more or less than 2000 inhabitants in the city), the socioeconomic status analysis is based on the standards of the education department (from 1: privileged to 4: underprivileged). There is no national data for the location, so the regional data were used.

### Data collection

#### Investigators

Examination of the children, adolescents and young adults with disability was conducted by 338 salaried dentists belonging to one of the three French national health insurance schemes. The investigator first conducted a structured interview with the child and his or her carer, and then examined the child. Any modification in the child's behavior or any declaration that suggested discomfort or distress led to the evaluation being interrupted. For the group of children without disability, the evaluation was conducted by 7 dentists who collected the data under these same conditions. All investigators underwent a one-day training course in their region prior to starting the study. The course consisted of three hours on the conditions for data collection and three hours on the psychosocial conditions of the population of children with disabilities.

#### Development of the list of indicators

A initial list of 36 indicators for examination was generated from literature review, interviews with professional experts in special care dentistry, in special education and with practitioners of the health insurance system. They were descriptions of global health, feeding, autonomy, access to dental care, oral hygiene, oral disease, and behavior. The feasibility of data collection and the clinical and social significance of the indicators were initially tested with a sample of 236 children and adolescents from 27 institutions distributed in 7 regions. Items resulting in a response rate of less than 70% were then deleted from the list that finally included 24 items. External consistency of the 24 item list was evaluated by comparing the prevalence of oral disease in the test group to the values founded in previous studies for different groups of children with disabilities. External consistency was good for food texture [21], [18], difficulties in pain communication [22], [23], halitosis [18] drooling [24], [25], presence of dental plaque, calculus and gingivitis [18], [26], [27], missing teeth [28], [29], fractured anterior tooth [30], caries decay [4], [27], [30], [31], dental infectious process [29], severe dysmorphology [32], and lack of compliance during oral examination [4], [33]. Cronbach's alpha was used to assess internal consistency of the list of 24 indicators for oral health evaluation. It was shown that the list was consistent (α = 0.93).

The inclusion criteria for children with disabilities were based on the diagnoses that justified children being orientated towards special schooling. These diagnoses were defined according to standard ICD-10 criteria [34].

#### Subject examination

Among the 24 item list, 20 items were objective indicators that were evaluated by the investigator during the oral examination. Children and adolescents in DC and DA groups were examined in the school dispensary in presence of their carer. Children in the NDC group were examined in their classroom in the presence of their teacher. For the evaluation of objective criteria, the investigators used disposable gloves, a mouth mirror and a portable light. Drying teeth and the use of a dental probe, both of which are indicated for caries and periodontal disease screening in the general population [35]–[37], were prohibited so as to avoid discomfort or injury. Four subjective indicators of oral health were obtained during a structured interview with the carer for each subject with disability, and with the child him or herself for each child without disability.

#### Stability of the indicators

Considering the vulnerability of the target population, it would have been unethical to build a true sample of children with disability for the calibration of the investigators. A virtual sample was therefore simulated with a set of 144 photos of the mouths of 6 to 20-year-old children and young adults with and without oral disease. The investigators had to evaluate the pictures twice, with a one-week interval.

Internal reliability was verified by paired comparisons of consistency between the two evaluations for all 144 pictures (intra-class correlation coefficients (ICC, r), and kappa coefficients (k)). Among the investigators who evaluated the children and adolescents with disabilities, 269 (79.6%) participated in the reliability test. Test-retest revealed that internal reliability was acceptable for the 144 pictures (mean ICC 95%, r = 0.65; k = 0.49, (p<0.001)). The distribution of the investigators within the range of the ICC values was: r>0.4 for 6 investigators (2.2%); 0.4<r<0.6 for 48 investigators (17.8%); 0.6<r<0.8 for 211 investigators (78.4%); and 0.8<r for 4 investigators (1.5%). External reliability was verified by testing the agreement between the criteria values given for each picture by the group of 269 investigators (ICC 95%, r = 0.63). All 7 investigators examining the group of children without disabilities participated in the reliability test. Test-retest showed that internal reliability was acceptable for the set of 144 pictures (mean 95% ICC, r = 0.74; k = 0.46) and that external reliability was good (95% ICC, r = 0.86).

#### Data analysis

Indicators of oral health were then associated to produce 3 original indices for global oral health. Initially a series of 3 algorithms were performed using logical association (If… then… else…) to rank the subjects according to the 4 levels of the Clinical Oral Health Index (COHI) (Table 2). A fourth algorithm was then used to rank the subjects according to the 4 levels of the Clinical Oral Care Needs Index (COCNI) (Table 3). Finally, preventive health or oral health education needs were determined by applying a fifth algorithm that ranked the subjects according to the two levels of the Clinical Oral Prevention Index (COPI) (Table 4).

**Table 2:** Conditions observed by algorithms ranking children among Clinical Oral Health Index values.

COHI Values		CRITERIA
**0**		**Having no criteria that had a medical impact:**
No oral health problems		No mucosal lesion on lips, tongue or jaw
		and Absence of dental plaque (Greene and Vermillon index = 0 for both arches) [35]
		and Absence of calculus (Greene and Vermillon index = 0 for both arches) [35]
		and No gingivitis (Loe and Silness index = 0 for both arches) [36]
		and No fractured anterior tooth
		and No missing anterior tooth
		and No missing posterior tooth
		and No dental caries
		and No infectious disease
		and No severe orofacial dysmorphology
		**AND having no criteria that had a social impact:**
		No halitosis
		and No drooling
		and No anterior tooth with a fractured, unrestored crown
1	**OR**	**Having no criteria that had a medical impact:**
Existence of one or more problems with a low to a moderate impact on health		No mucosal lesion on lips, tongue or jaw
		or Absence of dental plaque (Green and Vermillon index = 0 for both arches) [35]
		or Absence of calculus (Green and Vermillon index = 0 for both arches) [35]
		or No gingivitis (Loe and Silness index = 0 for both arches) [36]
		or No fractured anterior tooth
		or No missing anterior tooth
		or No missing posterior tooth
		or No dental caries
		or No infectious disease
		or No orofacial dysmorphology
		**AND having at least one criteria that had a social impact:**
		Presence of halitosis
		or Presence of drooling
		or At least one anterior tooth with a fractured unrestored crown
		**Having at least one criterion that had a medical impact**
		At least one mucosal lesion on lips, tongue or jaw
		or Dental plaque (Greene and Vermillon index >0, on a group of teeth or all the teeth of at least one arch) [35]
		or Calculus (Greene and Vermillon index >0, on a group of teeth or all the teeth of at least one arch) [35]
		or Localized gingivitis (Loe and Silness index >0 on a group of teeth or at least one arch) [36]
		or At least one anterior tooth with a fractured, unrestored crown
		or One limited anterior edentulous segment (1 or 2 anterior teeth)
		or At least one minor posterior edentulous segment (missing all molars and premolars on 1 or 2 half-arches with at least one residual inter-arch dental contact)
		or At least one incipient carious lesion, but no developed carious lesion (stage 1 or 2 according to the Eckstrand classification for carious lesions) [37]
		or Presence of a simple orofacial dysmorphology
		**… regardless of the criteria that had a social impact**
2		**Having at least one criteria that had an important medical impact**
Existence of one or more problems with important to severe impact on health		Generalized gingivitis (Loe and Silness index >0 for both arches) [35]
		or Missing at least 3 anterior teeth
		or At least one major posterior edentulous segment (missing all molar and premolars on at least 2 half-arches, without any residual inter-arch dental contact)
		or At least one developed carious lesion (stage 3 or 4 according to the Eckstrand classification for carious lesions) either on a deciduous or a permanent tooth [37]
		or At least one infected lesion (presence of an abscess, or a tooth with pulpal exposure, or a fistula)
		or Presence of complex orofacial dysmorphology
		**… regardless of the criteria that had a social impact**
**Undetermined**		At least one undetermined criteria that had a medical impact and regardless of the criteria that had a social impact.
Others conditions by elimination		or Having no criteria that had a medical impact but having at least one undetermined criteria that had a social impact.

**Table 3:** Conditions observed by algorithms ranking children among Clinical Oral Care Needs Index values.

COCNI Values	Suspected health conditions	CRITERIA
**3 **Urgent need for care or examination	Marker of local infectious disease	During the last three months the child expressed discomfort or pain in his/her mouth and consecutively he/she had no dental visit. or At least one mucosal lesion on lips, tongue or jaw or At least one infected lesion (presence of an abscess, or a tooth with pulp exposure cavity, or a fistula) AND absence of any systemic disease* requiring specific oral health monitoring.
	Marker of focal infectious disease	During the last three months the child expressed discomfort or pain in his/her mouth and consecutively he/she had no dental visit. or Presence of at least one mucosal lesion on lips, tongue or jaw or At least one infected lesion (presence of an abscess, or a tooth with a pulp exposure, or a fistula) or Presence of generalized gingivitis (Loe and Silness index >0 for both arches) AND Existence of a systemic disease requiring specific oral health monitoring*
	Marker of traumatic lesions	During the last three months the child expressed discomfort or pain in his/her mouth and consecutively he/she had no dental visit. or Presence of at least one mucosal lesion on lips, tongue or jaw
	Marker of oral disease with functional or social consequences	During the last three months the child expressed discomfort or pain with his/her mouth and consecutively he/she had no dental visit.
**2** Need for care or examination	Marker of local infectious disease	Presence of calculus or Presence of gingivitis or Presence of at least one anterior tooth with a fractured, unrestored crown or Presence of at least one developed carious lesion. AND Absence of a systemic disease* requiring specific oral health monitoring
	Marker of focal infectious disease	Presence of calculus. or Presence of a localized gingivitis or Presence of at least one anterior tooth with a fractured, unrestored crown or Presence of at least one developed carious lesion. AND Existence of a systemic disease* requiring specific oral health monitoring
	Marker of traumatic lesions	Presence of at least one anterior tooth with a fractured, unrestored crown
**1** Need for examination	Marker of local infectious disease	No dental visit over the last 12 months or Presence of dental plaque or Presence of at least one incipient carious lesion. AND Absence of a systemic disease* requiring specific oral health monitoring
	Marker of focal infectious disease	No dental visit over the last 12 months or Presence of dental plaque or Presence of at least one incipient carious lesion. AND Existence of a systemic disease* requiring specific oral health monitoring
	Marker of traumatic lesions	No dental visit over the last 12 months
	Marker of oral disease with functional or social consequences	No dental visit over the last 12 months or Presence of an anterior edentulous segment from 1 to 6 teeth on at least one arch. or Presence of a posterior edentulous segment for children up to 13 years old) or Presence of untreated severe orofacial dysmorphology
**0** No need for care nor examination		Other conditions by elimination

* Epilepsy, congenital cardiac disease, bronchopneumopathy (including asthma), internal prosthesis, immunodeficiency and hematological disease, or diabetes.

**Table 4:** Conditions observed by algorithms ranking children among Clinical Oral Prevention Index values.

COPI Values	CRITERIA
**1**	Existence of systemic disease requiring specific oral health monitoring*
Existence of at least one preventive or dental health education action need	or Presence of dental plaque
	or Lack of autonomy for feeding
	or Being fed by tube or parenteral nutrition
	or Restriction to puréed foods
	or Eating hyper-calorific food complements or drinking sweetened drinks
	or Coughing regularly during meals
	or Presence of halitosis
	or Having difficulties communicating pain
	or Drooling
	or Being uncooperative during oral examination [38]
**0**	
No need for either preventive health action or dental education	Other conditions, by elimination

* as defined in the descriptive results section.

The distributions of the NDC and DC groups across the various levels of the COHI, COCNI and COPI indices were compared to determine whether having a disability is a risk factor for poor oral health. The impact of age on oral health status was determined by comparing the distributions of the DC and DA groups across the different levels of the COHI, COCNI and COPI oral health indices. Odds ratios (OR) were calculated at 95% confidence intervals for inter-group comparisons.

The impact of 69 (untrained) out of 338 (trained) examiners for the DC and NDC group not participating in the test retest exercise, was evaluated by comparing the distributions of the subjects evaluated by untrained and trained examiners across the different levels of the COHI, COCNI and COPI oral health indices.

## Results

### Descriptive results

A total of 8,401 children, adolescents and young adults with disability were randomly selected among the 1,259 institutions that agreed to participate. Refusals to participate were declared in 332 cases, absence from the institution at the time of evaluation was recorded in 808 cases, and two children died before the investigator visit. Hence, 2,487 children aged 6 to 12 years old were included in the DC group (935 males, 552 females; mean age±SD: 9.75±1.88 years (95% CI = 9.68–9.82)), and 4,772 adolescents and young adults aged 13 to 20 years old were included in the DA group (2,920 males, 1,852 females; mean age±SD: 15.85±1.92 years (95% CI = 15.79–15.90)). Medical grounds for special schooling were categorized into 8 groups as follows: 1) any intellectual disability, regardless of severity (45.4%); 2) autism (4.6%), other psychological developmental problems (11.5%), schizophrenia and delusional disorders (2%), other mental and behavioral diseases (6.8%); 3) Down syndrome (9.5%) and other chromosome anomalies (2.4%); 4) developmental disorder (3.1%) and neonatal or maternal disease (3%); 5) epilepsy (1.9%) and other anomalies of the central nervous system (3%); 6) other diseases (3.9%); 7) unfavorable family and social background (1.6%); 8) missing data (0.5%). Systemic diseases requiring oral health monitoring were recorded in both the DC and DA groups (maximum 3 per subject). Epilepsy, congenital cardiac disease, bronchopneumopathy (including asthma), internal prosthesis, immunodeficiency and hematological disease, and diabetes, were recorded for 1027, 410, 287, 32, 32 and 31 subjects, respectively. Others miscellaneous systemic diseases were recorded in 408 cases. There were no distribution-based differences either in medical conditions or in gender between the DC and DA groups.

The NDC population consisted of 1,772 healthy children aged 6 to 12 years old from 22 mainstream schools. Among this group, refusals to participate were received from either parents or teachers of 71 children, evaluation failed for lack of coordination between school staff and investigators for another 56 children, and 4 children were absent from school at the time of evaluation. Hence, 1,641 children without disability aged between 6 and 12 year old were included (862 males, 779 females; mean age±SD: 7.98±1.61 years (95%CI = 7.91–8.06)). Gender and age distribution differed significantly between DC and NDC groups with both prevalence of boys (Chi Square test, p<0.001) and mean age being greater in the DC group than in NDC group (Chi Square test, p<0.01).

### Oral health status

Subject distributions in terms of oral health criteria and the indices are presented in tables 5 and 6, respectively. Distribution comparisons between the NDC and DC groups showed that more children with disability were rated COHI level 1 or 2 than level 0 (OR = 3.97; 95% CI = 3.25–4.86). Moreover, the number of children for whom some oral health indicators could not be estimated was greater in the DC group than in the NDC group (OR = 2.41; 95% CI = 1.74–3.32). Comparisons of COCNI distributions showed that more children with disability were in need of urgent care or examination (level 3) or non-urgent care (level 2) than those without disability (OR = 2.01; 95%CI = 1.77–2.28). COPI index distribution comparisons showed that more children with disability were in need of preventive oral health care or oral health education than children without disability (OR = 5.25, 95% CI = 4.55–6.02).

**Table 5:** Subject distribution among the oral health criteria for the three groups of children or adolescents.

Criteria for oral health	Children without disability (NDC group)			Children with disability (DC group)			Adolescents with disability (DA group)		
	6–12 yrs old			6–12 yrs old			13–20 yrs old		
	n	Yes	*95%CI*	n	Yes	*95%CI*	n	Yes	*95%CI*
Lack of autonomy for feeding	1,625	2.2%	*1.5–2.9*	2,487	18.6%	*17.1–20.1*	4,772	9.6%	*8.8–10.4*
Eating puréed foods	1,621	1.2%	*0.7–1.7*	2,466	8.4%	*7.3–9.5*	4,743	4.5%	*3.9–5.1*
Being fed by tube or parenteral nutrition	1,641	0.0%	*0*	2,487	0.8%	*0.5–1.2*	4,772	0.6%	*0.4–0.8*
Drinking sweetened drinks regularly	1,625	25.9%	*23.8–28.0*	2,483	11.9%	*10.7–13.2*	4,768	10.6%	*9.7–11.5*
Coughing often during meals	1,621	7.4%	*6.1–8.7*	2,188	31.9%	*30–33.9*	4,263	21.5%	*20.3–22.7*
Having recent discomfort or pain in the mouth	1,374	29.0%	*26.6–31.4*	2,330	7.5%	*6.4–8.9*	4,599	6.6%	*5.9–7.3*
Lack of a dental consultation consecutive - recent pain or discomfort*	401	42.0%	*37.2–46.8*	177	33.8%	*26.8–40.8*	188	57.8%	*50.7–64.9*
Difficulty expressing pain	1,366	15.8%	*13.9–17.7*	2,390	30.5%	*28.7–32.4*	4,664	20.6%	*19.4–21.8*
No dental visit over the last 12 months	1,277	52.5%	*49.8–55.2*	1,670	55.7%	*53.3–58.1*	3,560	55.7%	*54.1–57.3*
Presence of hali-sis	1,641	6.6%	*5.4–7.8*	2,465	6.0%	*5.1–6.9*	4,767	8.6%	*7.8–9.4*
Presence of drooling	1,641	0.0%	*0*	2,320	12.7%	*11.4–14.1*	4,599	8.4%	*7.6–9.2*
Presence of dental plaque	1,641	2.6%	*1.8–3.4*	2,376	11.5%	*10.2–12.8*	4,655	17.9%	*16.8–19*
Presence of calculus	1,631	1.5%	*0.9–2.1*	2,359	3.3%	*2.6–4*	4,644	6.0%	*5.3–6.7*
Presence of gingivitis	1,641	10.4%	*8.9–11.9*	2,358	36.5%	*34.6–38.4*	4,644	50.0%	*48.6–51.4*
Presenting at least one missing anterior -oth	1,641	0.0%	*0*	2,484	0.9%	*0.6–1.3*	4,766	1.0%	*0.7–1.3*
Presenting at least one missing posterior segment†	1,641	0.0%	*0*	2,484	0.3%	*0.1–0.5*	4,766	0.2%	*0.1–0.3*
Presence of at least one fractured anterior -oth left unres-red	1,620	0.9%	*0.4–1.4*	2,382	6.7%	*5.7–7.7*	4,671	8.1%	*7.3–8.9*
Presence of at least one incipient carious lesion ‡	1,641	12.7%	*11.1–14.3*	2,223	18.1%	*16.5–19.7*	4,525	24.3%	*23.1–25.6*
Presence of at least one developed carious lesion §	1,641	1.9%	*1.2–2.6*	2,223	9.4%	*8.2–10.6*	4,525	16.5%	*15.4–17.6*
Presence of at least one mucosal lesion on lips, -ngue or jaw	1,641	1.8%	*1.2–2.4*	2,366	8.9%	*5.3–12.5*	4,636	10%	*9.1–10.9*
At least one dental infectious process ∥	1,641	2.7%	*1.9–3.5*	2,297	9.3%	*8.1–10.5*	4,555	7.3%	*6.5–8.1*
Presence of severe orofacial dysmorphology **	1,611	37.6%	*35.2–40*	2,290	63.5%	*61.5–65.5*	4,631	58.1%	*56.7–59.5*
Lack of treatment for dysmorphology *	605	83.0%	*80–86*	1,454	94.1%	*92.9–95.3*	2,598	89.7%	*88.5–90.9*
Being uncooperative during oral examination††	1,641	10.5%	*9–12*	2,487	26.4%	*24.7–28.1*	4,772	15.8%	*14.8–16.8*

* for those having pain.

† absence of molars or premolars in at least one quarter of the mouth.

‡ stage 1 or 2 according to the Eckstrand classification for carious lesions without stage 3 or 4 caries, either on a deciduous or permanent tooth [37].

§ stage 3 or 4 according to the Eckstrand classification for carious lesions either on a deciduous or permanent tooth [37].

∥ presence of an abscess, or a tooth with pulp exposure, or a fistula related to dental disease.

** Four groups of malocclusion were defined: G1: Complete overjet of one or more than one tooth. G2: 1) Overjet of 6 mm or more than 6 mm; or 2) Labial or buccal openbite concerning three pairs of teeth or more than three; or 3) Significant or considerable crowding or overcrowding in labial or buccal arch sectors. G3: 1) Reverse overbite of at least one upper incisor or reverse overbite of at least one lower incisor; or 2) reverse overbite of post-canine teeth on one side or overbite of a complete or half arch buccal sector, provoking deflection or deviation of the mandible on contact through the intercuspal position or 3) overcoupled teeth. G4: 1) Deep overbite of front teeth associated with an overjet; or 2) Bilateral reverse overbite associated with an early deviating contact during the mandibular closing phase. Malocclusions of types G1 and G3 are considered as simple when not associated with malocclusions pertaining to G2 or G4. Malocclusions of types G2 and G4 and those associated with other malocclusions are considered as complex.

†† according to the modified Venham scale [38] score ≥1.

The modalities of subject distribution among the criteria were dichotomized for simplification. n: number of subjects for whom the criteria was evaluated; Yes: percentage of subjects experiencing the disorder.

**Table 6:** Distribution of the children with and without disabilities according to the values of the Clinical Oral Health Index (COHI), the Clinical Oral Care Needs Index (COCNI) and the Clinical Oral Prevention Index (COPI).

	Children without disability			Children with disability			Adolescents with disability		
	6–12 yrs old (NDC group)			6–12 yrs old (DC group)			13–20 yrs old (DA group)		
**Clinical Oral Health Index**	number	%	*95% CI*	number	%	*95% CI*	number	%	*95% CI*
0: no problem	357	21.8	*19.8–23.7*	157	6.3	*1.3–5.3*	88	1.8	*1.4–2.2*
1: at least one low to moderate problem	790	48.1	*45.7–50.5*	941	37.8	*35.9–39.7*	1 984	41.6	*40.2–42.9*
2: at least one important to severe problem	445	27.1	*24.9–29.3*	1 218	49.0	*45.3–50.9*	2 282	47.8	*46.3–49.2*
Unspecified level	49	3.0	*2.1–3.8*	171	6.9	*5.9–7.9*	418	8.8	*7.9–9.6*
*Total*	*1641*	*100%*		*2487*	*100%*		*4772*	*100%*	
**Clinical Oral Care Needs Index**	number	%	*95% CI*	number	%	*95% CI*	number	%	*95% CI*
0: No need for care nor examination	296	18.0	*16.4–19.8*	244	9.8	*8.6–10.9*	390	8.2	*7.4–8.9*
1: Need for examination	668	40.7	*37.6–42.3*	787	31.6	*29.7–33.4*	1123	23.5	*22.3–24.7*
2: Need for care	391	23.8	*21.7–25.8*	1013	40.7	*38.7–42.6*	2413	50.6	*48.6–52.0*
3: Urgent need for care or examination	286	17.4	*15.5–19.2*	443	17.8	*16.3–19.3*	846	17.7	*14.3–18.8*
*Total*	*1641*	*100%*		*2487*	*100%*		*4772*	*100%*	
**Clinical Oral Prevention Index**	number	%	*95% CI*	number	%	*95% CI*	number	%	*95% CI*
0: No need for prevention or dental education	1292	78.7.	*76.7–80.7*	1459	58.7	*56.8–60.7*	2297	48.1	*46.7–49.5*
1: Need for prevention or dental education	349	21.3	*19.3–23.3*	1028	41.3	*39.3–43.3*	2475	51.9	*50.4–53.3*
*Total*	*1641*	*100%*		*2487*	*100%*		*4772*	*100%*	

COHI index distribution comparisons between the DC and DA groups showed that a higher number of adolescents and young adults were rated COHI level 1 or 2 than level 0 (OR = 3.52; 95% CI = 2.7–4.6). Moreover, the number of subjects who could not be rated due to unspecified criteria was significantly higher in the DA group than in the DC group (OR = 1.3; 95% CI = 1.08–1.56). COCNI index distribution comparisons showed that more adolescents and young adults were in need of urgent care or examination (level 3) or non-urgent care (level ), whereas slightly more younger children were either in need of examination (level 1) or were not in need of care or examination (level 0) than children with disabilities (OR = 1.52; 95% CI = 1.38–1.69). COPI index distribution comparisons showed that adolescents and young adults with disabilities had greater needs for preventive oral health care or oral health education actions than children (OR = 1.53, 95% CI = 1.39–1.69).

A stratified comparison between the DC and NDC groups was performed testing for the impact of gender and age on child distribution between COHI, COCNI and COPI. It was shown that the distribution of children between the DC and NDC groups remained different regardless of gender (Chi Square test, p<0.001, for distribution in COHI, COCNI and COPI) and age for all age classes (Chi Square test, p<0.05 COHI, COCNI and COPI), except for the distribution of 7 to 8 year old children in COPI (Chi Square test, non significant).

The distributions of the children who did not participate in the evaluation for refusal for participation, absence from school or death did not differ between the group of 269 trained investigators and those 69 untrained investigators (Chi Square test, non significant). A total of 1343 subjects with disabilities were evaluated by the 69 untrained examiners (Table 7) The distributions across the different levels of COHI, COCNI and COPI of the 395 DC group children who were evaluated by the 69 untrained examiners did not differ from those of the other 2092 DC group children who were evaluated by the 269 trained examiners. The distributions across the oral health indices of the 948 adolescents in the DA group who were evaluated by the untrained examiners differ significantly from those of the 3824 other DA group children who were evaluated by the trained examiners for COHI (Chi square test, p<0.005) and COCNI (Chi square test, p<0.001). There were fewer subjects in both level 2 for COHI and level 3 for COCNI among the DA group subjects who were evaluated by the untrained examiners. The distribution across COPI did not differ significantly depending on whether the examiners were trained or not.

**Table 7:** Distribution of children and adolescents with disabilities (respectively DC and DA groups) across the values of the Clinical Oral Health Index (COHI), the Clinical Oral Care Needs Index (COCNI) and the Clinical Oral Prevention Index (COPI) dependant on the training of the examiners.

	DC GROUP					DA GROUP				
**Clinical Oral Health Index**	Trained Examiners (n = 269)		Untrained examiners (n = 69)		Chi Square	Trained Examiners (n = 269)		Untrained examiners (n = 69)		Chi Square
0: no problem	131	*6.26%*	26	*6.58%*	p = 0.35	64	*1.67%*	24	*2.53%*	p<0.05
1: at least one low to moderate problem	777	*37.14%*	164	*41.52%*		1594	*41.68%*	390	*41.14%*	
2: at least one important to severe problem	1043	*49.86%*	175	*44.30%*		1852	*48.43%*	430	*45.36%*	
Unspecified level	141	*6.74%*	30	*7.59%*		314	*8.21%*	104	*10.97%*	
Total	2092	*100%*	395	100%		3824	*100%*	948	*100%*	
**Clinical Oral Care Needs Index**	Trained Examiners (n = 269)		Untrained examiners (n = 69)		Chi Square	Trained Examiners (n = 269)		Untrained examiners (n = 69)		Chi Square
0: No need for care nor examination	199	*9.51.%*	45	*11.39%*	p = 0.10	292	*7.64%*	98	*10.34%*	p<0.01
1: Need for examination	670	*32.039%*	117	*29.62%*		882	*23.06%*	241	*25.42%*	
2: Need for care	836	*39.96%*	177	*44.81%*		1945	*50.86%*	468	*49.37%*	
3: Urgent need for care or examination	387	*18.50%*	56	*14.18%*		705	*18.44%*	141	*14.87%*	
Total	2092	*100%*	395	*100%*		3824	*100%*	948	*100%*	
**Clinical Oral Prevention Index**	Trained Examiners (n = 269)		Untrained examiners (n = 69)		Chi Square	Trained Examiners (n = 269)		Untrained examiners (n = 69)		Chi Square
0: No need for prevention or dental education	1231	*58.84%*	228	57.72%	p = 0.85	1853	*48.46%*	444	*46.84%*	p = 0.45
1: Need for prevention or dental education	861	*41.16%*	167	42.28%		1971	*51.54%*	504	*53.16%*	
Total	2092	*100%*	395	100%		3824	*100%*	948	*100%*	

Chi square test compared the subjects' distribution between subgroups.

## Discussion

This is the largest cross-sectional study to report oral health status for children and adolescents with disability. It demonstrates that the prevalence of poor oral health is increased in children with disability than in children without, and that this situation worsens with age. This study is also the first attempt to produce an original biopsychosocial indicator for oral health. The construct validity of the list of indicators could discriminate between subjects by age and presence of a disability. The concept of a clinical index generated by an algorithmic association of various clinical indicators is completely new in oral health, and offers a sensitive clinical measurement that could be used transversally in different populations.

Improving oral health is a specific concern for individuals with disability as oral health has both local and systemic consequences. Poor oral health is a factor for co-morbidity when associated with systemic disease. It increases the risk of infectious complications for patients presenting systemic diseases such as congenital cardiac disease, immunodeficiency or diabetes, or those with internal prostheses, and plays a direct role in the aggravation of chronic respiratory disease that is the main cause of mortality in people with disability [39]–[41]. For patients with epilepsy or mental deficiencies, both neurological and behavioral problems may be related to undiagnosed and untreated oral pain. This survey raises questions as to the measures that could be taken to decrease inequalities in oral health for children and adolescents with disability. There are three main strands to improving oral health in this population: improving oral hygiene, defining specific training for dental professionals, and allocating funds to cover dental care access and service utilization [42]. Oral hygiene decreases bacterial load by eliminating the lung pathogens present on the tongue, gum tissue and other oral mucosa or contained in dental plaque. Persons with disability, however, do not always have the neuromotor abilities required to independently and effectively perform oral hygiene [43]. Consequently, care staff and families who help with daily dental hygiene tasks should be targeted for educative programs [44], [45]. In some countries, including France, the healthcare system does not currently identify the professionals needed to teach care staff. Training of hygienists and tutoring special care issues during dental education could help to improve oral hygiene and care standards for people with disability [46].

There are both political and economic choices to be made before specific funds can be allocated for this population. It has been shown in the USA that children with the most limited functional ability were 50% less likely to meet the health insurance core outcome than those without limitations [47]. In France, the national health insurance system does not cover specific oral care procedures for people with disability, despite the fact that the State has declared its obligation to provide administrative facilitators to compensate for disability issues in the 2002 Disability Rights Bill. Other countries, including the USA, are examining the adoption of new standards and studying the continuing obstacles of limited government support for dental services [48].

There were many potential methodological biases that could undermine this study. The first question is related to the representativity of our sample against national populations of children with and without disabilities. The sample of children and adolescents with disabilities was randomly selected from a child population in 1279 out of 1325 special schools. In France, the vast majority of children with learning disabilities are schooled in institutions, following a decision by a special commission which evaluates their medical condition. Special schools therefore offer an ideal context in order to gain access to a large national sample of these children. It was however impossible to compare the sample of children and adolescents with disability to the national population of children with disabilities, as we had no information on the population from the 46 institutions that did not consent to participate in the study. In addition we have no demographic information on the population of children with severe disabilities who are schooled either at home, or in ordinary schools. This discrepancy in the constitution of samples cannot be controlled and could induce bias. It was however the only practical way to undertake the study, as it was impossible to constitute a wider sample of children with disabilities in the usual conditions that are respected for epidemiological studies. An even greater degree of difficulty was found in constituting a sample of adolescents and young adults with disabilities. Ethical, political, financial and technical difficulties were barriers that led to abandon the project of comparing the oral health conditions in DA group to those of a control sample.

Secondly, the methodology for the reliability tests was based on photograph-based evaluations rather than on clinical examinations. Compared to a subject based examination, the photos gave a partial view of the subject, reducing him/her to a part of his/her mouth or face. In these conditions, the quality of the computer screens that served for photo evaluation was a factor that might affect the external reliability, in addition to the psychometric characteristics of each individual investigator. Despite these conditions, internal and external reliability remained acceptable.

Thirdly, the examination/interview of children and adolescents with disability was conducted by 338 dentists working for a French national health insurance scheme while those without disability were examined/interviewed by 7 other dentists. In addition, the investigators were not isolated, and independent assessments for both groups cannot be expected. As these conditions could not be modified, we tried to control them by giving the same training to each group of investigators. In particular and in order to limit the psychological impact of contact with children suffering from severe disability on the investigators' evaluation, specific training was provided regarding patients with special needs prior to the survey.

Fourth, a total of 69 out 338 examiners did not participate in the test retest exercise. It was however demonstrated that this point did not alter the distribution across the COHI, COCNI and COPI indices in the DC group children but that it could affect the subjects distribution across COHI and COCNI for the DA group (Table 7). The untrained examiners who did not participate in the test-retest exercise underestimated the severity of the oral health disease and the needs for treatment in the group of adolescents with disabilities. Consequently it could be assumed that the differences between the DA and DC groups would be increased if all the examiners had completed the test-retest exercise.

Fifth, among the 24 indicators for examination/interviews, four (coughing often during meals, having recent discomfort or pain, difficulties expressing pain, and being uncooperative during oral examination) were subjectively evaluated using either care staff report for the DC and DA groups, or self report for the NDC groups. The information thus compiled might not be exactly the same in these different conditions. For this reason, these subjective indicators were not included in the items determining the COHI and COCNI indices but featured exclusively in the COPI index.

Despite these limitations, the COHI, COCNI and COPI indices offer sensitive clinical measurements of oral health status in epidemiological surveys. Further developments are needed to increase the field of application of these biopsychosocial indices for oral health.
